# A Hybrid Deep Learning and Visualization Framework for Pushing Behavior Detection in Pedestrian Dynamics

**DOI:** 10.3390/s22114040

**Published:** 2022-05-26

**Authors:** Ahmed Alia, Mohammed Maree, Mohcine Chraibi

**Affiliations:** 1 Institute for Advanced Simulation, Forschungszentrum Jülich, 52425 Jülich, Germany; a.alia@fz-juelich.de; 2 Computer Simulation for Fire Protection and Pedestrian Traffic, Faculty of Architecture and Civil Engineering, University of Wuppertal, 42285 Wuppertal, Germany; 3 Department of Management Information Systems, Faculty of Engineering and Information Technology, An-Najah National University, Nablus, Palestine; 4 Department of Information Technology, Faculty of Engineering and Information Technology, Arab American University, Jenin, Palestine

**Keywords:** deep learning, convolutional neural network, EfficientNet-B0-based classifier, image classification, crowd behavior analysis, pushing behavior detection, motion information maps, deep optical flow

## Abstract

Crowded event entrances could threaten the comfort and safety of pedestrians, especially when some pedestrians push others or use gaps in crowds to gain faster access to an event. Studying and understanding pushing dynamics leads to designing and building more comfortable and safe entrances. Researchers—to understand pushing dynamics—observe and analyze recorded videos to manually identify when and where pushing behavior occurs. Despite the accuracy of the manual method, it can still be time-consuming, tedious, and hard to identify pushing behavior in some scenarios. In this article, we propose a hybrid deep learning and visualization framework that aims to assist researchers in automatically identifying pushing behavior in videos. The proposed framework comprises two main components: (i) Deep optical flow and wheel visualization; to generate motion information maps. (ii) A combination of an EfficientNet-B0-based classifier and a false reduction algorithm for detecting pushing behavior at the video patch level. In addition to the framework, we present a new patch-based approach to enlarge the data and alleviate the class imbalance problem in small-scale pushing behavior datasets. Experimental results (using real-world ground truth of pushing behavior videos) demonstrate that the proposed framework achieves an 86% accuracy rate. Moreover, the EfficientNet-B0-based classifier outperforms baseline CNN-based classifiers in terms of accuracy.

## 1. Introduction

In entrances of large-scale events, pedestrians either follow the social norm of queuing or force some pushing behavior to gain faster access to the events [1]. Pushing behavior in this context is an unfair strategy that some pedestrians use to move quickly and enter an event faster. This behavior involves pushing others and moving forward quickly by using one’s arms, shoulders, elbows, or upper body, as well as using gaps among crowds to overtake and gain faster access [2,3]. Pushing behavior, as opposed to queuing behavior, can increase the density of crowds [4]. Consequently, such behavior may lead to threatening the comfort and safety of pedestrians, resulting in dangerous situations [5]. Thus, understanding pushing behavior, what causes it, and the consequences are crucial, especially when designing and constructing comfortable and safe entrances [1,6]. Conventionally, researchers have attempted to study pushing behavior manually by observing and identifying pushing cases among video recordings of crowded events. For instance, Lügering et al. [3] proposed a rating system on forward motions in crowds to understand when, where, and why pushing behavior appears. The system relies on two trained observers to classify the behaviors of pedestrians over time in a video (the behavior is classified into either pushing or non-pushing categories). In this context, each category includes two gradations: mild and strong for pushing, and falling behind and just walking for non-pushing. For more details on this system, we refer the reader to [3]. To carry out their tasks, the observers analyzed top-view video recordings using pedestrian trajectory data and PeTrack software [7]. However, this manual rating procedure is time-consuming, tedious, and requires a lot of effort by observers, making it hard to identify pushing behavior, specifically when the number of videos and pedestrians in each video increase [3]. Consequently, there is a pressing demand to develop an automatic and reliable framework to identify when and where pushing behavior appears in videos. This article’s main motivation is to help social psychologists and event managers identify pushing behavior in videos. However, automatic pushing behavior detection is highly challenging due to several factors, including diversity in pushing behavior, the high similarity and overlap between pushing and non-pushing behaviors, and the high density of crowds at event entrances.

According to a computer vision perspective, automatic pushing behavior detection belongs to the video-based abnormal human behavior detection field [8]. Several human behaviors have been addressed, including walking in the wrong direction [9], running away [10], sudden people grouping or dispersing [11], human falls [12], suspicious behavior, violent acts [13], abnormal crowds [14], hitting, pushing, and kicking [15]. It is worth highlighting that pushing as defined in [15] is different from the “pushing behavior” term in this article. In [15], pushing is a strategy used for fighting, and the scene contains only up to four persons. To the best of our knowledge, no previous studies have automatically identified pushing behavior for faster access from videos.

With the rapid development in deep learning, CNN has achieved remarkable performance in animal [16,17] and human [13,18] behavior detection. The main advantage of CNN is that it directly learns the useful features and classification from data without any human effort [19]. However, CNN requires a large training dataset to build an accurate classifier [20,21]. Unfortunately, this requirement is unavailable in most human behaviors. To alleviate this limitation, several studies have used a combination of CNN and handcrafted feature descriptors [22,23]. The hybrid-based approaches use descriptors to extract valuable information. Then, CNN automatically models abnormal behavior from the extracted information [24,25]. Since labeled data for pushing behavior are scarce, the hybrid-based approaches could be more suitable for automatic pushing behavior detection. Unfortunately, the existing approaches are inefficient for pushing behavior detection [22]. Their main limitations are: (1) their descriptors do not work well to extract accurate information from dense crowds due to occlusions, or they cannot extract the needed information for pushing behavior representation [22,26]; (2) Some used CNN architectures are not efficient enough to deal with the high similarity between pushing and non-pushing behaviors (high inter-class similarity) and the increased diversity in pushing behavior (intra-class variance), leading to misclassification [25,26].

To address the above limitations, we propose a hybrid deep learning and visualization framework for automatically detecting pushing behavior at the patch level in videos. The proposed framework exploits video recordings of crowded entrances captured by a top-view static camera, and comprises two main components: (1) motion information extraction aims to generate motion information maps (MIMs) from the input video. A MIM is an image that contains useful information for pushing behavior representation. This component divides each MIM into several MIM patches, making it easier to see where pedestrians are pushing. For this purpose, recurrent all-pairs field transforms (RAFT) [27] (one of the newest and most promising deep optical flow methods) and the wheel visualization method [28,29] are combined; (2) The pushing patch annotation adapts the EfficientNet-B0-based CNN architecture (the EfficientNet-B0-based CNN  [30] is an effective and simple architecture in the EfficientNet family proposed by Google in 2019, achieving the highest accuracy in the ImageNet dataset [31]) to build a robust classifier, which aims to select the relevant features from the MIM patches and label them into pushing and non-pushing categories. We utilized a false reduction algorithm to enhance the classifier’s predictions. Finally, the component outputs pushing the annotated video showed when and where the pushing behaviors appeared.

We summarize the main contributions of this article as follows:

1. To the best of our knowledge, we proposed the first framework dedicated to automatically detecting when and where pushing occurs in videos.
2. An integrated EfficientNet-B0-based CNN, RAFT, and wheel visualization within a unique framework for pushing behavior detection.
3. A new patch-based approach to enlarge the data and alleviate the class imbalance problem in the used video recording datasets.
4. To the best of our knowledge, we created the first publicly available dataset to serve this field of research.
5. A false reduction algorithm to improve the accuracy of the proposed framework.

The rest of this paper is organized as follows: Section 2 reviews the related work of video-based abnormal human behavior detection. In Section 3, we introduce the proposed framework. A detailed description of dataset preparation is given in Section 4. Section 5 discusses experimental results and comparisons. Finally, the conclusion and future work are summarized in Section 6.

## 2. Related Works

Existing video-based abnormal human behavior detection methods can be generally classified into object-based and holistic-based approaches [25,26]. Object-based methods consider the crowd as an aggregation of several pedestrians and rely on detecting and tracking each pedestrian to define abnormal behavior [32]. Due to occlusions, these approaches face difficulties in dense crowds [33,34]. Alternatively, holistic-based approaches deal with crowds as single entities. Thus, they analyze the crowd itself to extract useful information and detect abnormal behaviors [24,25,34]. In this section, we briefly review some holistic-based approaches related to the context of this research. Specifically, the approaches are based on CNN or a hybrid of CNN and handcrafted feature descriptors.

Tay et al. [35] presented a CNN-based approach to detect abnormal actions from videos. The authors trained the CNN on normal and abnormal behaviors to learn the features and classification. As mentioned before, this type of approach requires a large dataset with normal and abnormal behaviors. To address the lack of large datasets with normal and abnormal behaviors, some researchers applied a one-class classifier using datasets of normal behaviors. Obtaining or preparing a dataset with only normal behaviors is easier than a dataset with normal and abnormal behaviors [34,36]. The main idea of the one-class classifier is to learn from the normal behaviors only; to define a class boundary between the normal and not defined (abnormal) classes. Sabokrou et al. [36] utilized a new pre-trained CNN to extract the motion and appearance information from crowded scenes. Then, they used a one-class Gaussian distribution to build the classifier from datasets with normal behaviors. In the same way, the authors of [34,37] used datasets of normal behaviors to develop their one-class classifiers. Xu et al. used a convolutional variational autoencoder to extract features in [34]. Then, multiple Gaussian models were employed to predict abnormal behavior. Ref.  [37] adopted a pre-trained CNN model for feature extraction and a one-class support vector machines to predict abnormal behavior. In another work, Ilyas et al. [24] used pre-trained CNN along with a gradient sum of the frame difference to extract relevant features. Afterward, three support vector machines were trained on normal behavior to detect abnormal behavior. In general, the one-class classifier is popular when the abnormal behavior or target behavior class is rare or not well-defined [38]. In contrast, the pushing behavior is well-defined and not rare, especially in high-density and competitive scenarios. Moreover, this type of classifier considers the new normal behavior as abnormal.

In order to overcome the drawback of CNN-based approaches and one-class classifier approaches, several studies used a hybrid-based approach with a multi-class classifier. Duman et al. [22] employed the classical Farnebäck optical flow method [23] and CNN to identify abnormal behavior. The authors used Farnebäck and CNN to extract the direction and speed information. Then, they applied a convolutional long short-term memory network for building the classifier. In [39], the authors used a histogram of gradient and CNN to extract the relevant features, while a least-square support vector was employed for classification. In a similar line of the hybrid approaches, Direkoglu [25] combined the Lucas–Kanade optical flow method and CNN to extract the relevant features and detect “escape and panic behaviors”. Almazroey et al. [26] employed mainly a Lucas–Kanade optical flow, pre-trained CNN, and feature selection (neighborhood component analysis) methods to select the relevant features. The authors then applied a support vector machine to generate a trained classifier. Zhou et al. [40] presented a CNN method for detecting and localizing anomalous activities. The study integrated optical flow with a CNN for feature extraction and it used a CNN for the classification task.

In summary, hybrid-based approaches have shown better accuracy than CNN-based approaches on small datasets [41]. Unfortunately, the reviewed hybrid-based approaches are inefficient for dense crowds and pushing behavior detection due to (1) their feature extraction parts being inefficient for dense crowds; (2) The reviewed approaches cannot extract all of the required information for pushing behavior representation; (3) Their classifiers are not efficient enough toward pushing behavior detection. Hence, the proposed framework combines the power of supervised EfficientNet-B0-based CNN, RAFT, and wheel visualization methods to solve the above limitations. The RAFT method works well for estimating optical flow vectors from dense crowds. Moreover, the integration of RAFT and wheel visualization helps to simultaneously extract the needed information for pushing behavior representation. Finally, the adapted EfficientNet-B0-based binary classifier detects distinct features from the extracted information and identifies pushing behavior at the patch level.

## 3. The Proposed Framework

**Figure 1:**
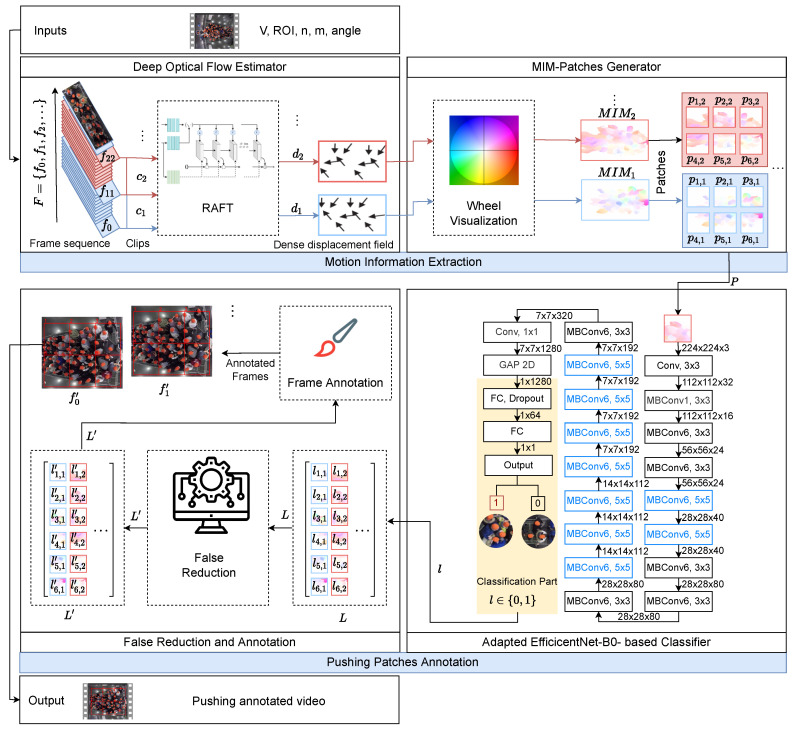
The architecture of the proposed automatic deep learning framework. *n* and *m* are two rows and three columns, respectively, for patching. Clip size *s* is 12 frames. MIM: motion information map. *P*: patch sequence. *L*: a matrix of all patches labels. $L^{\prime }$: an updated *L* by false reduction algorithm. *V*: the input video. ROI: region of interest (entrance area). angle: the rotation angle of the input video.

This section describes the proposed framework for automatic pushing behavior detection at the video patch level. As shown in Figure 1, there are two main components: motion information extraction and pushing patches annotation. The first component extracts motion information from input video recordings, which is further exploited by the pushing patch annotation component to detect and localize pushing behavior, producing pushing annotated video. The following subsections discuss both components in more detail.

### 3.1. Motion Information Extraction

This component employs RAFT and wheel visualization to estimate and visualize the crowd motion from the input video at the patch level. The component has two modules, a deep optical flow estimator and a MIM patch generator.

The deep optical flow estimator relies on RAFT to calculate the optical flow vectors for all pixels between two frames. RAFT was introduced in 2020; it is a promising approach for dense crowds because it reduces the effect of occlusions on optical flow estimation [27]. RAFT is based on a composition of CNN and recurrent neural network architectures. Moreover, RAFT has strong cross-dataset generalization and its pre-trained weights are publicly available. For additional information about RAFT, we refer the reader to [27]. This module is based on the RAFT architecture with its pre-trained weights along with three inputs, which are a video of crowded event entrances, the rotation angle of the input video, and the region of interest (ROI) coordinates. To apply RAFT, firstly, we determine the bounding box of the entrance area (ROI) in the input video *V*. This process is based on user-defined left–top and bottom–right coordinates of the ROI in the pixel unit. Then, we extract the frame sequence $F=\{f_{t}\;|\;t=1,2,3,\ldots ,T\}$ with ROI only from *V*, where $f_{t}\in R^{w\times h\times 3}$, *w* and *h* are the $f_{t}$ width and height, respectively, 3 is the number of channels, *t* is the order of the frame *f* in *V*, and *T* is the total number of frames in *V*. After that, we rotate the frames (based on the user-defined angle) in *F* to meet the baseline direction of the crowd flow that is used in the classifier, which is from left to right. The rotation process is essential to improve the classifier accuracy because the classifier will be built by training the adapted EfficientNet-B0 on the crowd flow from left to right. Next, we construct from *F* the sequence of clips $C=\{c_{i}\;|\;i=1,2,3,\ldots \}$ and $c_{i}$ is defined as

(1) $$c_{i}=\left\{f_{(i-1)\times (s-1)+1},f_{(i-1)\times (s-1)+2},\ldots ,f_{(i-1)\times (s-1)+s}\right\},$$

where *s* is the clip size. Finally, RAFT is applied on $c_{i}$, to calculate the dense displacement field $d_{i}$ between $f_{(i-1)\times (s-1)+1}$ and $f_{(i-1)\times (s-1)+s}$. The output of RAFT of each pixel location 〈x,y〉 in $c_{i}$ is a vector, as shown in.

(2) $${\langle u_{\langle x,y\rangle },v_{\langle x,y\rangle }\rangle }_{c_{i}}=RAFT\left({\langle x,y\rangle }_{c_{i}}\right),$$

where *u* and *v* are horizontal and vertical displacements of a pixel at the 〈x,y〉 location in $c_{i}$, respectively. This means $d_{i}$ is a matrix of the vector values for the entire $c_{i}$, as described in

(3) $$d_{i}={\left\{{\langle u_{\langle x,y\rangle },v_{\langle x,y\rangle }\rangle }_{c_{i}}\right\}}_{\left(x,y)=(1,1\right)}^{\left(w,h\right)}$$

In summary, $d_{i}$ is the output of this module and will act as the input of the MIM patch generator module.

**Figure 2:**
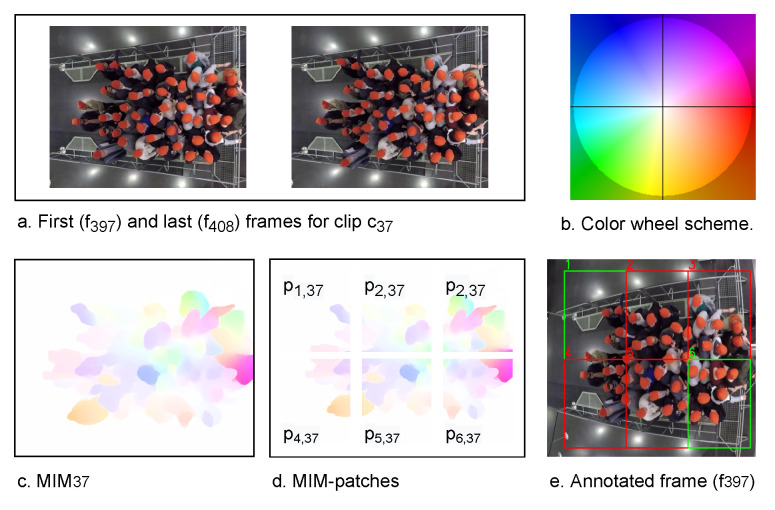
An illustration of two frames (experiment 270 [42]), color wheel scheme [29], MIM, MIM patches, and annotated frame. In sub-figure (**e**), red boxes refer to pushing patches, while green boxes represent non-pushing patches.

The second module, the MIM patch generator, employs the wheel visualization to infer the motion information from each $d_{i}$. Firstly, the wheel visualization calculates the magnitude and the direction of each motion vector at each pixel 〈x,y〉 in $d_{i}$. Equations (4) and (5) are used to calculate the motion direction and magnitude, respectively. Then, from the calculated information, the wheel visualization generates $MIM_{i}$, where $MIM_{i}\in R^{w\times h\times 3}$. In MIM, the color refers to the motion direction and the intensity of the color represents the motion magnitude or speed. Figure 2 shows the color wheel scheme (b) and an example of MIM ($MIM_{37}$) (c) that is generated from $c_{37}$, whose first and last frames are $f_{397}$ and $f_{408}$, respectively (a). $c_{37}$ is taken from the experiment 270 [42].

(4) $$\theta {\left(\langle x,y\rangle \right)}_{c_{i}}=\pi^{-1}\arctan\left(\frac{v_{\langle x,y\rangle }}{u_{\langle x,y\rangle }}\right)$$

(5) $$mag{\left(\langle x,y\rangle \right)}_{c_{i}}=\sqrt{u_{\langle x,y\rangle }^{2}+v_{\langle x,y\rangle }^{2}}$$

To detect pushing behavior at the patch level, the MIM patch generator divides each $MIM_{i}$ into several patches. A user-defined row (*n*) and column (*m*) are used to split $MIM_{i}$ into patches $\left\{p_{k,i}\in R^{(w/m)\;\times \;(h/n)\;\times \;3}\;|\;k=1,2,\ldots ,n\times m\;\right\}$, where *k* is the order of the patch in $MIM_{i}$. Afterward, each $p_{k,i}$ is resized to a dimension of 224 × 224 × 3, which is the input size of the second component of the framework. For example, $MIM_{37}$ in Figure 2c represents an entrance with dimensions 5 × 3.4 m on the ground, and it is divided into 2 × 3 patches $\left\{p_{k,37}\;|\;k\leq 6\right\}$ as given in Figure 2d. These patches are equal in pixels, whereas the area that is covered by them is not necessarily equal. The far patches from the camera cover a larger viewing area compared to close patches; because the far-away object has fewer pixels per m than a close object [43]. In Figure 2d, the average width and height of the $p_{k,37}$ are approximately 1.67 × 1.7 m.

In summary, the output of the motion information extraction component can be described as $P=\{p_{k,i}\in R^{224\;\times \;224\;\times \;3}\;|\;k\leq n\times m\;\&\;i\leq |C|\}$, and will serve as input for the second component of the framework.

### 3.2. Pushing Patches Annotation

This component localizes the pushing patches in $c_{i}\in C$, annotates the patches in the first frame ($f_{(i-1)\times (s-1)+1}$) of each $c_{i}$, and stacks the annotated frame sequence $F^{\prime }=\{f_{i}^{\prime }\;|\;i=1,2,\ldots ,|C|\}$ as a video. The Adapted EfficientNet-B0-based classifier and false reduction algorithm are the main modules of this component. In the following, we provide a detailed description.

The main purpose of the first module is to classify each $p_{k,i}$∈P as pushing or non-pushing. The module is based on EfficientNet-B0 and real-world ground truth of pushing behavior videos. Unfortunately, the existing effective and simple EfficientNet-B0 is unsuitable for detecting pushing behavior because its classification is not binary. However, binary classification is required in our scenario. Therefore, we modify the classification part in EfficientNet-B0 to support a binary classification. The module in Figure 1 shows the architecture of the adapted EfficientNet-B0. Firstly, it executes a 3 × 3 convolution operation on the input image with dimensions of 224 × 224 × 3. Afterwards, the next 16 mobile inverted bottleneck convolutions are used to extract the feature maps. The final stacked feature maps $\in R^{7\times 7\;\times \;1280}$, where 7 and 7 are the dimensions of each feature map, and 1280 is the number of feature maps. The following global average pooling2D (GAP) layer reduces the dimensions of the stacked feature maps into 1×1×1280. For the binary classification, we employed a fully connected (FC) layer with a ReLU activation function and a dropout rate of 0.5 [44] before the final FC. The final layer operates as output with a sigmoid activation function to find the probability δ of the class of each $p_{k,i}$∈P.

In order to generate the trained classifier, we trained the adapted EfficientNet-B0 with pushing and non-pushing MIM patches. The labeled MIM patches were extracted from a real-world ground truth of pushing behavior videos, where the ground truth was manually created. In Section 4 and Section 5.1, we show how to prepare the labeled MIM patches and train the classifier, respectively. Overall, after several empirical experiments (Section 5.2), the trained classifier on MIM patches of 12 frames produces the best accuracy results. Therefore, our framework uses 12 frames for the clip size (*s*). Moreover, the classifier uses the threshold for determining the label $l_{k,i}$ of the input $p_{k,i}$ as:

(6) $$l_{k,i}=\left\{\begin{array}{cc} 1\;(\mathrm{pushing}\;\mathrm{class}) & \mathrm{if}\;\delta \geq 0.5\\ 0\;(\mathrm{non}-\mathrm{pushing}\;\mathrm{class}) & \mathrm{if}\;\delta <0.5 \end{array}\right.$$

Finally, the output of this module can be described as $L=\{l_{k,i}\in 0,1\;|\;k\leq n\times m\;\&\;i\leq |C|\}$ and will perform as the input of the next module.

In the second module, the false reduction algorithm aims to reduce the number of false predictions in *L*, which improves the overall accuracy of the proposed framework. Comparing the predictions (*L*) with the ground truth pushing, we notice that the time interval of the same behavior of each patch region could help improve the accuracy of the framework. We assume a threshold value of $\frac{34}{25}$ second. This value is based on visual inspection.

**Figure 3:**
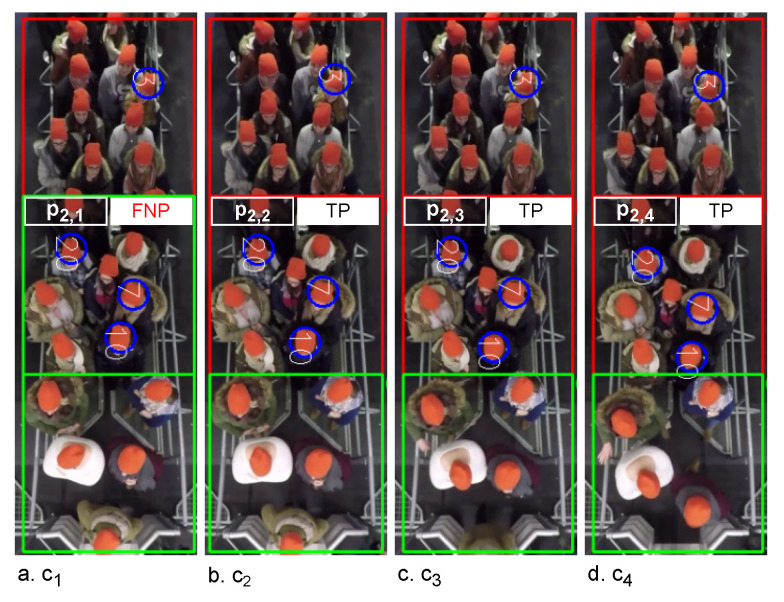
Examples of the visualized classifier predictions with ground truth pushing. The images represent the first frames $\left\{f_{1},f_{12},f_{23},f_{34}\right\}$ of $\left\{c_{1},c_{2},c_{3},c_{4}\right\}$ in a video, respectively; the video is for experiment 110 [42]. Red boxes: pushing patches. Green boxes: non-pushing patches. Blue circles: ground truth pushing. FNP: false non-pushing. TP: true pushing.

The example in Figure 3 visualizes the $\left\{l_{k,i}\;|\;k\leq 3\;\&\;i\leq 4\right\}$ on the first frame of $c_{1},c_{2},c_{3}$, and $c_{4}$ in the video. Each $c_{i}$ represents $\frac{12}{25}$ second. $c_{1}$ (Figure 3a) contains one false non-pushing, $p_{2,1}$, while the same region of the patch in $\left\{c_{2},c_{3},c_{4}\right\}$ is true pushing (Figure 3b–d). This means, we have two time intervals for $\left\{p_{2,i}\;|\;i\leq 4\right\}$. The first has one clip ($c_{1}$) (Figure 3a) with a duration of $\frac{12}{25}$ second, which is lesser than the defined threshold. The second time interval contains three clips ($\left\{c_{2},c_{3},c_{4}\right\}$), with durations equal to the threshold. Then the algorithm changes the prediction of $p_{2,1}$ to “pushing”, while it confirms the predictions of $p_{2,2},p_{2,3}$, and $p_{2,4}$. Algorithm 1 presents the pseudocode of the false reduction algorithm. Lines 2–8 show how to reduce the false predictions of the patches in $\left\{c_{i}\;|\;i\leq |c|-2\right\}$ Then, lines 9–16 recheck the first two clips ($c_{1},c_{2}$) to discover the false predictions that are not discovered by lines 2–8. After that, lines 17–32 focus on the last two clips $\left\{c_{|C|-1},c_{\left|C\right|}\right\}$. Finally, the updated *L* is stored in $L^{\prime }$, which can be described as $L^{\prime }=\{l_{k,i}^{\prime }\in 0,1\;|\;k\leq n\times m\;\&\;i\leq |C|\}$.

After applying the false reduction algorithm, the pushing patch annotation component based on $L^{\prime }$ identifies the regions of pushing patches on the first frame for each $c_{i}$ to generate the annotated frame sequence $F^{\prime }$. Finally, all annotated frames are stacked as a video, which is the final output of the proposed framework.
**Algorithm 1** False Reduction.**Input:**     matrix[N,M]←L**Output:**     $L^{\prime }$1: **for** i←0,1,…,N **do**
2:    **for** j←0,1,…,M−2 **do**
        ▹ Excepting the last two clips3:        **if** matrix[i, j] ≠ matrix[i, j+1] **then**4:           **if** count(matrix[i, j] in matrix[i, j+2 to j+4]) > 1 **then**5:               matrix[i, j+1] ← not matrix[i, j+1]6:           **end if**7:        **end if**8:    **end for**
        ▹ Recheck the first two clips9:    **if** matrix[i, 0 to 2] is not identical **then**10:        **if** matrix[i, 1] is not in matrix[i, 2 to 4] **then**11:           matrix[i, 1] ← not matrix[i, 1]12:        **end if**13:        **if** matrix[i, 0] not in matrix[i, 1 to 3] **then**14:           matrix[i, 0] ← not matrix[i, 0]15:        **end if**16:    **end if**
          ▹ For the last two clips17:    **if** matrix[i, M−1] ≠ matrix[i, M−2] **then**18:        **if** matrix[i, M−1] ≠ matrix[i, M−3] **then**19:           matrix[i, M−1] ← not matrix[i, M−1]20:        **end if**21:    **end if**22:    **if** matrix[i, M−1] ≠ matrix[i, M−2] **then**23:        **if** matrix[i, M−1] = matrix[i, M−3] **then**24:           matrix[i, M−2] ← not matrix[i, M−2]25:        **end if**26:    **end if**27:    **if** matrix[i, M−1] = matrix[i, M−2] **then**28:        **if** matrix[i, M−1] not in matrix[i, M−5 to M−3] **then**29:           matrix[i, M−1] ← not matrix[i, M−1]30:           matrix[i, M−2] ← not matrix[i, M−2]31:        **end if**32:    **end if**33: **end for**34: $L^{\prime }\leftarrow matrix$

## 4. Datasets Preparation

This section prepares the required datasets for training and evaluating our classifier. In the following, firstly, four MIM-based datasets are prepared. Then, we present a new patch-based approach for enlarging the data and alleviating the class imbalance problem in the MIM-based datasets. Finally, the patch-based approach is applied to the datasets.

### 4.1. MIM-Based Datasets Preparation

**Figure 4:**
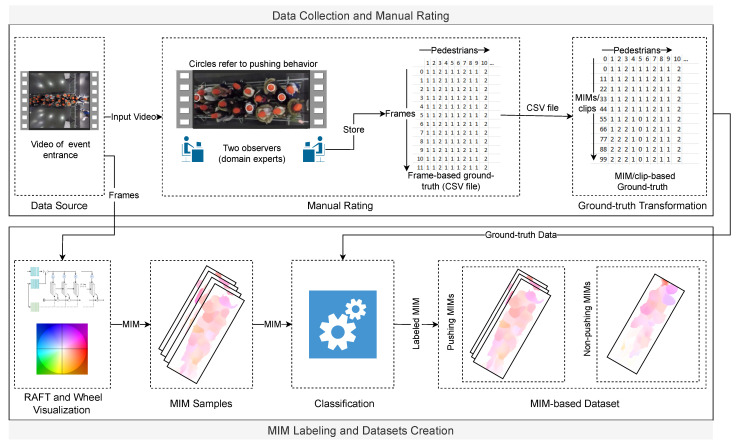
The pipeline of MIM-based dataset preparation.

In this section, we prepare four MIM-based datasets using two clip sizes, Farnebäck and RAFT optical flow methods. Two clip sizes (12 and 25 frames) are used to study the impact of the period of motion on the classifier accuracy. Selecting a small clip size (*s*) for the MIM sequence (MIM${}^{Q_{s}}$) leads to redundant and irrelevant information, while a large size leads to a few samples. Consequently, we chose 12 and 25 frames as the two clip sizes. The four datasets can be described as RAFT-MIM${}^{Q_{12}}$, RAFT-MIM${}^{Q_{25}}$, Farnebäck-MIM${}^{Q_{12}}$, and Farnebäck-MIM${}^{Q_{25}}$. For more clarity, the “RAFT-MIM${}^{Q_{12}}$” term means that a combination of RAFT and wheel visualization is used to generate the MIM${}^{Q_{12}}$. As mentioned before, the EfficientNet-B0 learns from MIM sequences generated based on RAFT. Therefore, RAFT-MIM${}^{Q_{12}}$-based and RAFT-MIM${}^{Q_{25}}$-based datasets play the primary role in training and evaluating the proposed classifier. Moreover, we create Farnebäck-MIM${}^{Q_{12}}$-based and Farnebäck-MIM${}^{Q_{25}}$-based datasets to evaluate the impact of RAFT on the classifier accuracy. The pipeline for preparing the datasets (Figure 4) is illustrated below.

#### 4.1.1. Data Collection and Manual Rating

**Figure 5:**
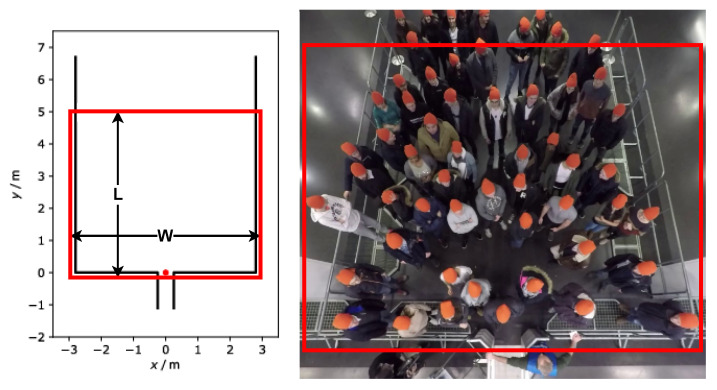
ROI in the entrance. (**Left**) experimental setup with the red dot indicating the coordinate origin [42], (**right**) overhead view of an exemplary experiment. The original frame in the right image is from [42]. The entrance gate width is 0.5 m. The rectangle indicates the entrance area (ROI). *L*: length of ROI in m. According to the experiment, the width of the ROI (w) varies from 1.2 to 5.6 m.

In this section, we discuss the data source and the manual rating methodology for the datasets. Five experiments were selected from the data archive hosted by the Forschungszentrum Jülich under CC Attribution 4.0 International license [42]. The experiments mimicked the crowded event entrances. The videos were recorded by a top-view static camera with a frame rate of 25 frames per second and 1920 × 1440 pixels resolution. In addition to the videos, parameters for video undistortion and trajectory data are available. In Figure 5, the left part sketches the experimental setup and Table 1 shows the different characteristics of the selected experiments.

Experts performing the manual rating are social psychologists who developed the corresponding rating system [3]. PeTrack [7] was used to track each pedestrian one-by-one, over every frame in the video experiments. Pedestrian ratings are annotated for the first frame when the respective participant becomes visible in the video. The first rating can be extended to the whole video and every frame if that pedestrian does not change his/her behavior. If there is a behavioral change during the experiment, then the rating is also changed. Likewise, it can be extended to the rest of the frames if there is no additional change in the behavior. The rating process is finished after every frame is filled with ratings for every pedestrian. The behaviors of pedestrians are labeled with numbers ∈{0,1,2}; 0 indicates that a corresponding pedestrian does not appear in the clip, while 1 and 2 represent non-pushing and pushing behaviors, respectively. Two ground truth files (MIM${}^{Q_{12}}$ and MIM${}^{Q_{25}}$) for each experiment were produced for this paper. Further information about the manual rating can be found in [3].

#### 4.1.2. MIM Labeling and Dataset Creation

Three steps are required to create the labeled MIM-based datasets. In the first step, we generated the samples from the videos; the samples were: RAFT-MIM${}^{Q_{12}}$, RAFT-MIM${}^{Q_{25}}$, Farnebäck-MIM${}^{Q_{12}}$, and Farnebäck-MIM${}^{Q_{25}}$ sequences. The MIM represents the crowd motion in the ROI, which is presented by the rectangle in Figure 5. It is worth mentioning that the directions of the crowd flows in the videos are not similar. This difference could influence building an efficient classifier because changing the direction is one candidate feature for pushing behavior representation. To address this problem, we unified the direction in all videos from left to right before extracting the samples. Additionally, to improve the efficiency of the datasets, we discarded roughly the first seconds from each video to guarantee that all pedestrians started to move forward.

Based on the ground truth files, the second step labels MIMs in the four MIM sequences into pushing and non-pushing. Each MIM that contains at least one pushing pedestrian is classified as pushing; otherwise, it is labeled as non-pushing.

Finally, we randomly split each dataset into three distinct sets: 70% for training, 15% for validation, and 15% for testing. The 70%-15%-15% split ratio is one of the most common ratios in the deep learning field [45]. The information about the number of pushing and non-pushing samples in the training, validation and test sets for the four MIM-based datasets is given in Table 2. As can be seen from Table 2, our MIM-based datasets suffer from two main limitations: lack of data and a class imbalance problem, since less than 20% of samples are non-pushing.

### 4.2. The Proposed Patch-Based Approach

**Figure 6:**
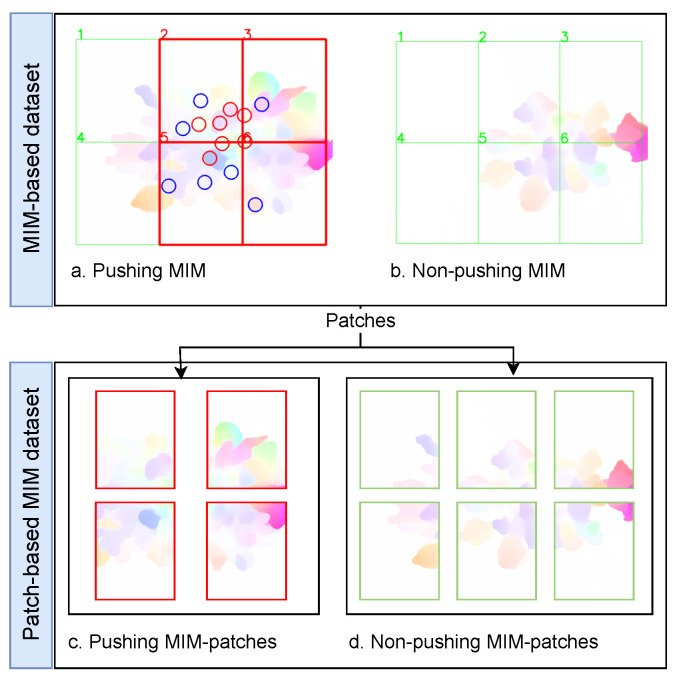
A simple example of the patch-based approach idea. Circles: ground truth pushing. Red boxes: pushing patches. Green boxes: non-pushing patches.

**Figure 7:**
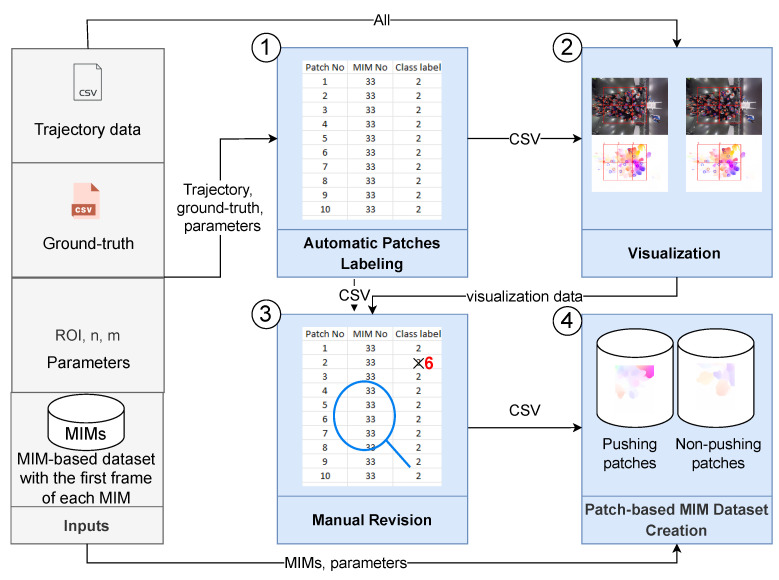
The flow diagram of the proposed patch-based approach. n and m: the numbers of rows and columns, respectively, that are used to divide ROI into n × m regions.

In this section, we propose a new patch-based approach to alleviate the limitations of the MIM-based datasets. The general idea behind our approach is to enlarge the small pushing behavior dataset by dividing each MIM into several patches. After that, we label each patch into “pushing” or “non-pushing” to create a patch-based MIM dataset. The patch should cover a region that can contain a group of pedestrians, where the motion information of the group is essential for pushing behavior representation. Section 5.2 investigates the impact of the patch area on the classifier accuracy. To further clarify the idea of the proposed approach, we take an example of a dataset with one pushing MIM and one non-pushing MIM, as depicted in Figure 6. After applying our idea with 2 × 3 patches on the dataset, we obtain a patch-based MIM dataset with four pushing, six non-pushing, and two empty MIM patches. The empty patches are discarded. In conclusion, the dataset is enlarged from two images into ten images. The methodology of our approach, as shown in Figure 7 and Algorithm 2, consists of four main phases: automatic patches labeling, visualization, manual revision, and patch-based MIM dataset creation. The following paragraphs discuss the inputs and the workflow of the approach.

Our approach relies on four inputs (Algorithm 2 and Figure 7, inputs part): (1) MIM-based dataset, which contains a collection of MIMs with the first frame of each MIM; the frames are used in the visualization phase; (2) ROI, *n* and *m*, parameters that aim to identify the regions for patches; (3) Pedestrian trajectory data to find the pedestrians in each patch; (4) Manual rating information (ground truth file) helps to label the patches.

**Figure 8:**
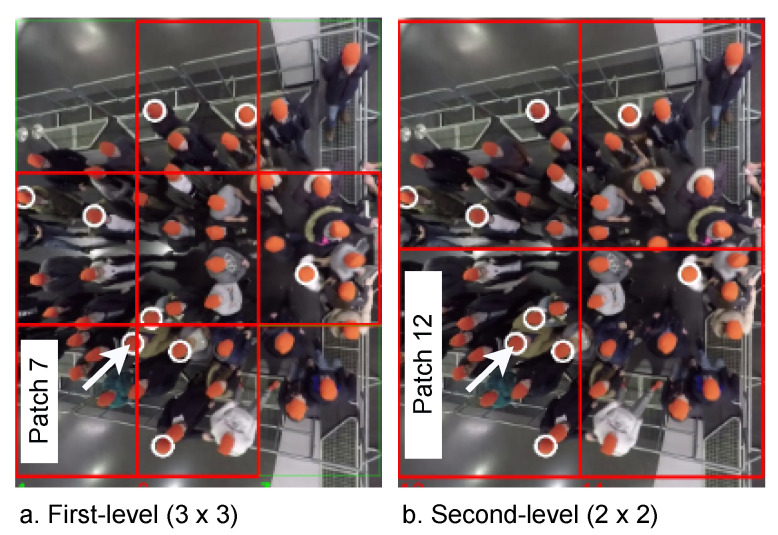
An example of identifying patches and the visualization process. The original frames are from [42]. Red boxes: pushing patches. Green boxes: non-pushing patches. White circles: ground truth pushing.

The first phase, automatic patch labeling, identifies and labels the patches in each MIM (Algorithm 2, lines 1–33 and Figure 7, first phase). The phase contains two steps: (1) Finding the regions of the patches. For this purpose, we find the coordinates of the regions that are generated from dividing the ROI area into n×m parts. The extracted regions can be described as $\left\{a_{k}|\;k=1,2,\ldots ,n\times m\right\}$, where $a_{k}$ represents a patch sequence $\left\{p_{k,i}\in R^{(w/m)\;\times \;(h/n)\;\times \;3}\;|\;i=1,2,\ldots ,|MIM^{Q}|\right\}$, *w* and *h* are the ROI width and height, respectively, see Algorithm 2, lines 1–15. We should point out that identifying the regions is performed on at least two levels; to avoid losing any useful information. For example, in Figure 8, we first split ROI by 3 × 3 regions (Algorithm 2, lines 2–8), while in the second level, we reduce the number of regions (2 × 2) to obtain larger patches (Algorithm 2, lines 9–15) containing the missing pushing behaviors (pushing behaviors are divided between the patches) in the first level; (2) Labeling the patches is executed according to the pedestrians’ behavior in each patch $p_{k,i}$. Firstly, we find all pedestrians who appear in $MIM_{i}$ (Algorithm 2, lines 18 and 19). Then, we label each $p_{k,i}$ as pushing if it contains at least one pushing behavior; otherwise, it is labeled as non-pushing (Algorithm 2, lines 20–28). Finally, we store k,i, and the label of $p_{k,i}$ in a CSV-file (Algorithm 2, lines 29 and 30).
**Algorithm 2** Patch-Based Approach.**Inputs:****dataset** ← collection of MIMs with the first frame of each MIM**ROI**←matrix[left_top:[x_coordinate,y_coordinate],right_bottom:[x_coordinate,y_coordinate]]n,m ← the numbers of rows and columns that are used to divide ROI into n×m regions.**trajectory** ← CSV file, each row represents $\langle order\;of\;\;frame\left(f_{t}\right),pedestrian\;no.,pixel\;x-coordinate,pixel\;y-coordinate\rangle$**ground_truth** ← CSV file, each row represents $\langle orderof\;c_{i}\;or\;MIM,behavior\;of\;pedestrian\;1,behavior\;of\;pedestrian\;2,\ldots ,$ behavioroflastpedestrian〉**Outputs:**pushing_folder, non-pushing_folder1:region←matrix[[]]▹ Automatic patches labeling2: patch_width←(ROI[1,0]−ROI[0,0])/m3: patch_height←(ROI[1,1]−ROI[0,1])/n4: **for** i←0,1,…,n−1 **do**5:    **for** j←0,1,…,m−1 **do**6:        region.append([ROI[0,0]+j×patch_width,ROI[0,1]+i×patch_height,ROI[0,0]+(j+1)×patch_width,ROI[0,1]+(i+1)×patch_height])7:    **end for**8: **end for**9: patch_width←(ROI[1,0]−ROI[0,0])/(m−1)10: patch_height←(ROI[1,1]−ROI[0,1])/(n−1)11: **for** i←0,1,…,n−2 **do**12:    **for** j←0,1,…,m−2 **do**13:        region.append([ROI[0,0]+j×patch_width,ROI[0,1]+i×patch_height,ROI[0,0]+(j+1)×patch_width,ROI[0,1]+(i+1)×patch_height])14:    **end for**15: **end for**16: file←CSVfile17: **for each** MIM∈dataset **do**18:    frame_order←MIMname19:    ped←Filter(trajectory.frame_order)[1]20:    patch_no←121:    **for each** patch_region∈region **do**22:        behavior←1 //non-pushing23:        **for each** ped∈patch_region **do**24:           **if** Filter(ground_truth.frame_order&ped)==2 **then**25:               behavior←2 //pushing26:               break27:           **end if**28:        **end for**29:        record←[patch_no,frame_order,behavior]30:        file.write(record)31:        patch_no←patch_no+132:    **end for**33: **end for**
▹ Visualization34: **for each** frame∈dataset **do**35:    frame_order←framename36:    ped←Filter(trajectory.frame_order)[1]37:    **for each** person∈ped **do**38:        behavior←Filter(ground_truth.frame_order&person)39:        **if** behavior ==2 **then**40:           draw a circle around the position 〈person[2],person[3]]〉 of pedestrian person[1] over frame41:        **end if**42:    **end for**43:    **for** patch_no←1,2,…,len(region) **do**44:        **if** Filter(file.frame_order&patch_no)[2]==2 **then**45:           draw a red rectangle around region[patch_no−1] over frame46:        **else**47:           draw a green rectangle around region[patch_no−1] over frame48:        **end if**49:    **end for**50: **end for**
▹ Manual revision51: **for each** frame∈dataset **do**52:    **for each** patch_region∈region **do**53:        manual revision of patch_region in frame54:        **if** patch_regioncontainsonlyapartofonepushingbehavioranditslabelis2 **then**55:           manually updating the label of the patch_region in file to 6, where 6 means unknown patch56:        **end if**57:    **end for**58: **end for**
▹ Patch-based MIM dataset creation59: **for each** MIM∈dataset **do**60:    MIM_order←MIMname61:    **for** patch_no←1,2,…,len(region) **do**62:        patch←MIM[region[patch_no−1,1]:region[patch_no−1,3],[region[patch_no−1,0]:region[patch_no−1,2]]63:        **if** Filter(file.MIM_order&patch_no)[2]==2 **then**64:           save patch to pushing_folder under name “MIM_order−patch_no"65:        **else if** Filter(file.MIM_order&patch_no)[2]==1 **then**66:           save patch to non-pushing_folder under name “MIM_order−patch_no"67:        **end if**68:    **end for**69: **end for**

Despite the availability of the pedestrian trajectories, the automatic patch labeling phase is not 100% accurate, affecting the quality of the dataset. The automatic way fails to label some of the patches that only contain a part of one pushing behavior. Therefore, manual revision is required to improve the dataset quality. To ease this process and make it more accurate, the visualization phase (Algorithm 2, lines 34–50 and Figure 7, second phase) visualizes the ground truth pushing (Algorithm 2, lines 36–42), and the label of each $p_{k,i}$ (Algorithm 2, lines 43–49) on the first frame of $MIM_{i}$. Figure 8 is an example of the visualization process.

The manual revision phase ensures that each $p_{k,i}$ takes the correct label by manually revising the visualization data (Algorithm 2, lines 51–58 and Figure 7, third phase). The criteria used in the revision are as follows: if $p_{k,i}$ only has a part of one pushing behavior, we change the labels to unknown labels in the CSV-file generated by the first phase; otherwise, the label of $p_{k,i}$ is not changed. The unknown patches do not offer complete information about pushing behavior or non-pushing behavior. Therefore, the final phase in our approach will discard them. A good example of an unknown patch is patch 7, Figure 8a. This patch contains a part of one pushing behavior, as highlighted by the arrow. On the other hand, patch 12 in the aforementioned example (b) contains the whole pushing behavior that we lose in discarding patch 7.

In the final phase (Algorithm 2, lines 59–69 and Figure 7, fourth phase), the patch-based MIM dataset creation is responsible for creating the labeled patch-based MIM dataset, containing two groups of MIM patches, pushing and non-pushing. Firstly, we crop $p_{k,i}$ from $MIM_{i}$ (Algorithm 2, line 62). Next, and according to the labels of the patches, the pushing patches are stored in the first group (Algorithm 2, lines 63 and 64), while the second group archives the non-pushing patches (Algorithm 2, lines 65 and 66).

### 4.3. Patch-Based MIM Dataset Creation

In this section, we aimed to create several patch-based MIM datasets using the proposed patch-based approach and the MIM-based datasets. The main purposes of the created datasets are: (1) to build and evaluate our classifier; (2) examine the influence of the patch area and clip size on classifier accuracy.

**Figure 9:**
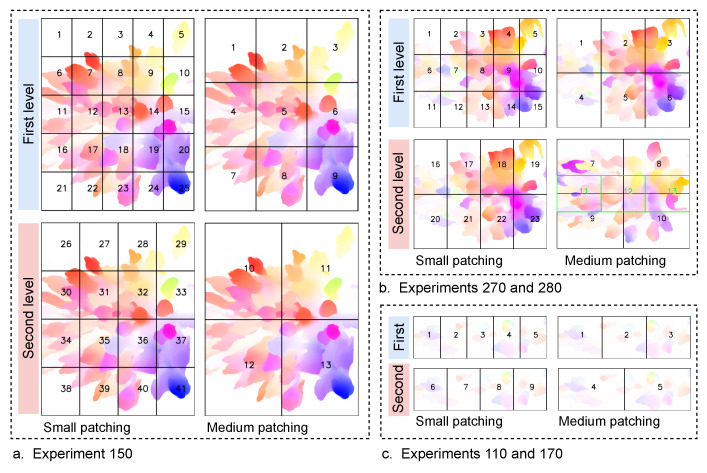
The visualization of patching for the experiments. Numbers represent the patch order in each experiment and level.

In order to study the impact of the patch area on classifier accuracy, we used two different areas. As we mentioned before, the regions covered by the patches should be enough to house a group of pedestrians. Therefore, according to the ROIs of the experiments, we selected the two patch areas as follows: 1 m × (1 to 1.2) m and 1.67 m × (1.2 to 1.86) m. The dimensions of each area refer to the length x width of patches. Due to the width difference between the experiment setups, there is a variation in the width between the experiments. Table 1 shows the width of each experiment’s setup, while the length of the ROI area in all experiment setups was 5 m (Figure 5, left part). For the sake of discussion, we name the 1 m × (1 to 1.2) m patch area as the small patch, and 1.67 m × (1.2 to 1.86) m as the medium patch. Moreover, the small and medium patching with the used levels are illustrated in Figure 9.

**Figure 10:**
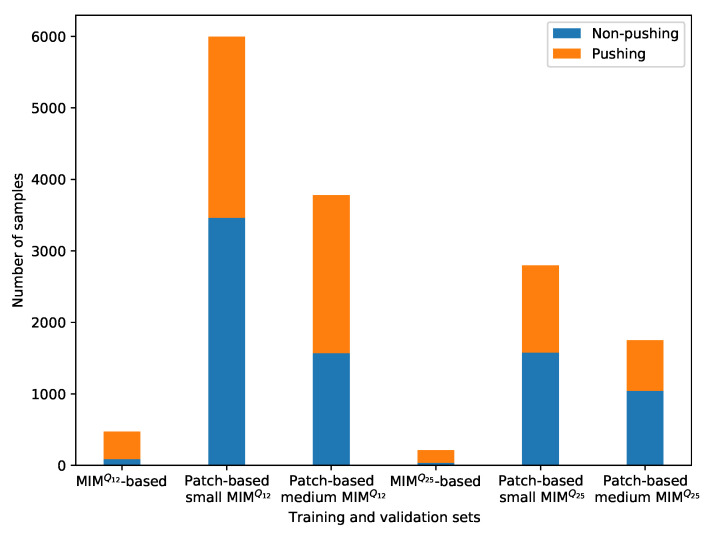
The visualization of the number of pushing and non-pushing samples for the training and validation sets.

The patch-based approach is performed on the RAFT-MIM-based training sets to generate patch-based RAFT-MIM training sets, while it creates patch-based RAFT-MIM validation sets from the RAFT-MIM-based validation sets. The created patch-based RAFT-MIM datasets with their numbers of labeled samples are presented in Table 3. The table and Figure 10 demonstrate that the proposed approach enlarges the RAFT-MIM-based training and validation sets in both small and medium patching. The approach roughly duplicates the MIM-based training and validation sets 13 times in small patching. While in medium patching, each MIM-based training and validation set is duplicated 8 times. Moreover, our approach decreases the class imbalance issue significantly.

The approach reduces the difference percentage between the pushing and non-pushing classes in the patch-based MIM training and validation sets as follows: patch-based small RAFT-MIM${}^{Q_{12}}$, from 62% to 16%. Patch-based medium RAFT-MIM${}^{Q_{12}}$, from 62% to 17%. Patch-based small RAFT-MIM${}^{Q_{25}}$, from 65% to 13%. Patch-based medium RAFT-MIM${}^{Q_{25}}$, from 65% to 20%.

Despite these promising results, we can only assess the efficiency of our approach when the CNN-based classifier is trained and tested on our patch-based RAFT-MIM datasets. For this important process, we generate four patch-based RAFT-MIM test sets. The patch-based approach applies the first level of patching on RAFT-MIM-based test sets (Table 2) to generate the patch-based RAFT-MIM test sets. We apply the first level in the small and medium patching (because we need to evaluate our classifier for detecting pushing behavior at the small and medium patches). Table 4 shows the number of labeled MIM patches in the patch-based RAFT-MIM test sets and their experiments. In Section 5.3, we discuss the impact of the patch-based approach on the accuracy of CNN-based classifiers.

## 5. Experimental Results

This section presents the parameter setup and performance metrics used in the evaluation. Then, it trains and evaluates our classifier and studies the impact of the patch area and clip size on the classifier performance. After that, we investigate the influence of the patch-based approach on the classifier performance. Next, the effect of RAFT on the classifier is discussed. Finally, we evaluate the performance of the proposed framework on the distorted videos.

### 5.1. Parameter Setup and Performance Metrics

For the training process, the RMSProp optimizer with a binary cross-entropy loss function was used. The batch size and epochs were set to 128 and 100, respectively. Moreover, when the validation accuracy did not increase for 20 epochs, the training process was automatically terminated. In the RAFT and Farnebäck methods, we used the default parameters.

The implementations in this paper were performed on a personal computer running the Ubuntu operating system with an Intel(R) Core(TM) i7-10510U CPU @ 1.80 GHz (8 CPUs) 2.3 GHz and 32 GB RAM. The implementation was written in Python using PyTorch, Keras, TensorFlow, and OpenCV libraries.

In order to evaluate the performance of the proposed framework and our classifier, we used accuracy and F1 score metrics. This combination was necessary since we had imbalanced datasets. Further information on the evaluation metrics can be found in [46].

### 5.2. Our Classifier Training and Evaluation, the Impact of Patch Area and Clip Size

In this section, we have two objectives: (1) training and evaluating the adapted EfficientNet-B0-based classifier. (2) Investigating the impact of the clip size and patch area on the performance of the classifier.

We compare the adapted EfficientNet-B0-based classifier with three well-known CNN-based classifiers (MobileNet [47], InceptionV3 [48], and ResNet50 [49]) to achieve the above objectives. The classification part in the well-known CNN architectures is modified to be binary. The four classifiers train from scratch on the patch-based RAFT-MIM training and validation sets. Then we evaluate the trained classifiers on patch-based RAFT-MIM test sets to explore their performance.

**Figure 11:**
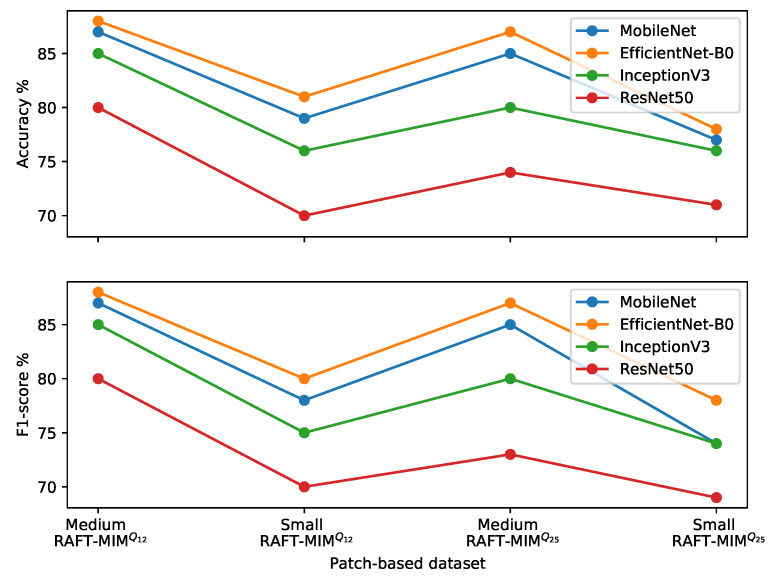
Comparisons of four classifiers over all patch-based RAFT-MIM sets.

From the results in Table 5 and Figure 11, it is seen that our trained classifier on the patch-based medium RAFT-MIM${}^{Q_{12}}$ dataset achieves better accuracy and F1 scores than other classifiers. More specifically, the EfficientNet-B0-based classifier has 88% accuracy and F1 scores. Furthermore, the medium patches help all classifiers to obtain better performances than small patches. At the same time, MIM${}^{Q_{12}}$ is better than MIM${}^{Q_{25}}$ for training the four classifiers in terms of accuracy and F1 score.

The patch area influences the classifier performance significantly. For example, medium patches improve the EfficientNet-B0-based classifier accuracy and F1 scores by 7% and 8%, respectively, compared to the small patches. On the other hand, the effect of the MIM sequence (clip size) on the classifier performance is lesser than the influence of the patch area. Compared to medium MIM${}^{Q_{25}}$, medium MIM${}^{Q_{12}}$ enhances the accuracy and F1 score by 1% in the EfficientNet-B0-based classifier.

In summary, the trained adapted EfficientNet-B0-based classifier on the patch-based medium RAFT-MIM${}^{Q_{12}}$ dataset achieves the best performance.

### 5.3. The Impact of the Patch-Based Approach

We evaluated the impact of the proposed patch-based approach on the performance of the trained classifiers on patch-based medium RAFT-MIM${}^{Q_{12}}$ training and validation sets. To achieve that, we trained the four classifiers on RAFT-MIM${}^{Q_{12}}$-based training and validation sets (Table 2). Then the trained classifiers were evaluated on patch-based medium RAFT-MIM${}^{Q_{12}}$ test sets (Table 4).

**Figure 12:**
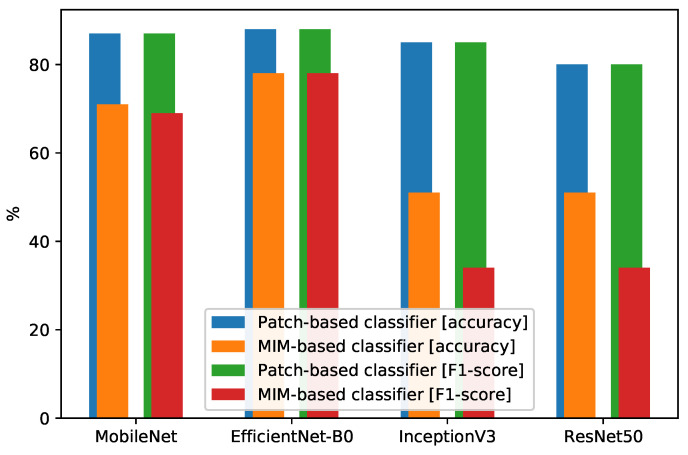
Comparison between MIM-based classifiers and patch-based classifiers.

Table 6 represents the performance of MIM-based classifiers. The comparison between patch-based classifiers and MIM-based classifiers is visualized in Figure 12. We can see that the EfficientNet-B0-based classifier (MIM-based classifier) achieves the best performance, which is a 78% accuracy and F1 score. In comparison, the corresponding patch-based classifier achieves an 88% accuracy and F1 score. This means that the patch-based approach improves the accuracy and F1 score of the EfficientNet-B0-based classifier by 10%. Similarly, in other classifiers, the patch-based approach increases the accuracy and F1 score by at least 15% for each.

### 5.4. The Impact of RAFT

**Figure 13:**
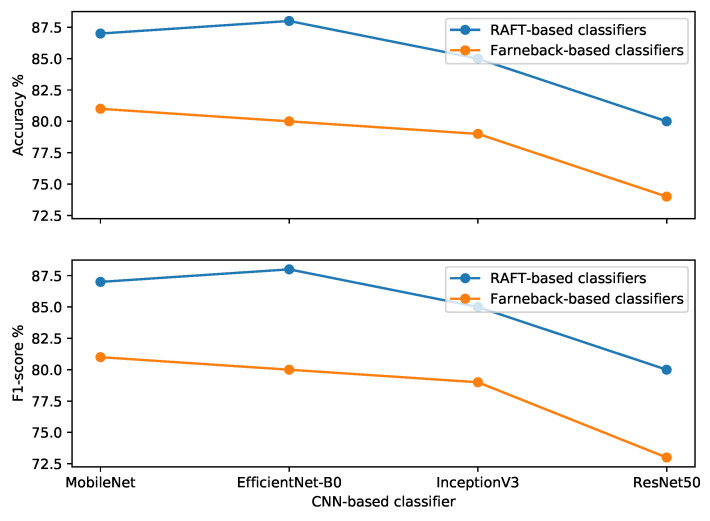
Comparison between the RAFT-based classifier and the Farnebäck-based classifier.

In order to study the impact of RAFT on our classifier, we trained it using the patch-based medium Farnebäck-MIM${}^{Q_{12}}$ dataset. Farnebäck is one of the most popular optical flow methods used in human action detection. Firstly, we created patch-based medium training and validation and test sets from the Farnebäck-MIM${}^{Q_{12}}$-based dataset (Table 2). The training and validation sets were used to train the EfficientNet-B0-based classifier (Farnebäck-based classifier), while the test set was used to evaluate the classifier. Finally, we compared the performance of the classifier based on RAFT with the classifier based on Farnebäck. As shown in Table 7 and Figure 13, we find that RAFT improves the classifier performance in all classifiers compared to Farnebäck. In particular, RAFT enhances the EfficientNet-B0-based classifier performance by 8%.

### 5.5. Comparison between the Proposed Classifier and the Customized CNN-Based Classifiers in Related Works

In this section, we evaluate our classifier by comparing it with two of the most recent customized CNN architectures (CNN-1 [25] and CNN-2 [35]) in the video-based abnormal human behavior detection field. Customized CNNs have simple architectures; CNN-1 used 75 × 75 pixels as an input image, three convolutional layers followed by batch normalization and max pooling operations. Finally, a fully connected layer with a softmax activation function was employed for classification. On the other hand, CNN-2 resized the input images into 28 × 28 pixels, then employed three convolutional layers with three max pooling layers (each max pooling layer with strides of 2 pixels). Moreover, it used two fully connected layers for predictions; the first layer was based on a ReLU activation function, while the second layer used a softmax activation function. For more details on CNN-1 and CNN-2, we refer the reader to [25,35], respectively.

**Figure 14:**
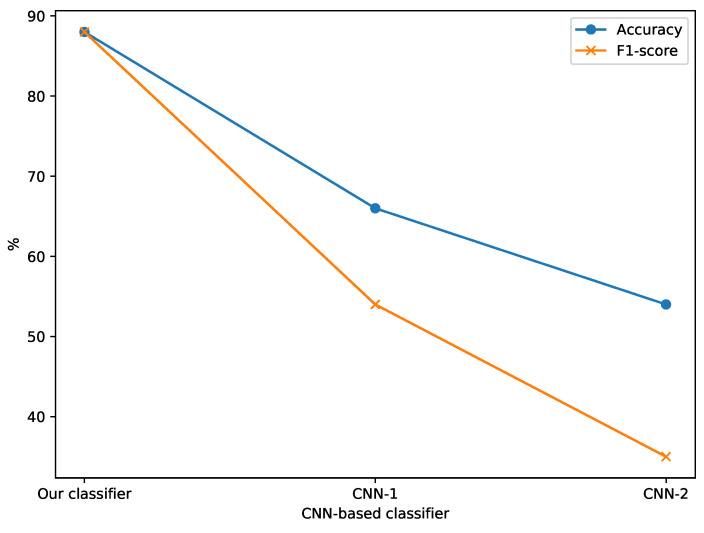
Comparison between our classifier, CNN-1 [25] and CNN-2 [35] based on the patch-based medium RAFT-MIM${}^{Q_{12}}$ dataset.

The three classifiers were trained and evaluated based on the patch-based medium RAFT-MIM${}^{Q_{12}}$ dataset. As shown in Table 8 and Figure 14, CNN-1 and CNN-2 obtained low accuracy and F1 scores (less than 61%), while our classifier achieved an 88% accuracy and F1 score.

**Figure 15:**
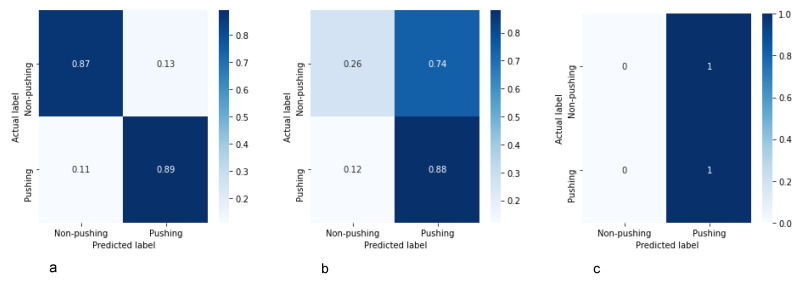
Confusion matrices for our classifier (**a**), CNN-1 [25] (**b**) and CNN-2 [35] (**c**) based on the patch-based medium RAFT-MIM${}^{Q_{12}}$ dataset.

In summary, and according to Figure 15, the reviewed customized CNN architectures are simple and not enough to detect pushing behaviors because the differences between pushing and non-pushing behaviors are not clear in many cases. To address this challenge, we need an efficient classifier (such as the proposed classifier).

### 5.6. Framework Performance Evaluation

Optical imaging systems often suffer from distortion artifacts [50]. According to [51], distortion is “a deviation from the ideal projection considered in a pinhole camera model, it is a form of optical aberration in which straight lines in the scene do not remain straight in an image”. The distortion leads to inaccurate trajectory data [52]. Therefore, PeTrack corrects the distorted videos before extracting the accurate trajectory data, whereas the required information for the correction is not often available. Unfortunately, training our classifier on undistorted videos could decrease the framework performance on distorted videos. Therefore, in this section, we evaluated the proposed framework performance on the distorted videos and studied the impact of the false reduction algorithm on the framework performance. To achieve both goals, firstly, we evaluated the framework’s performance without the algorithm on the distorted videos. Then, the framework with the algorithm was evaluated. Finally, we compared both performances.

**Figure 16:**
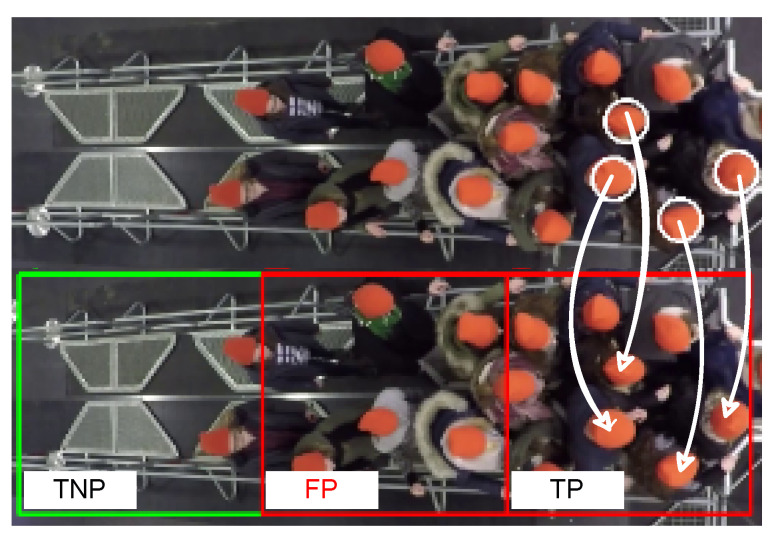
An example of the used qualitative methodology. (**Top**) the first frame of an undistorted clip; (**Bottom**) the first frame of a distorted clip. White arrows: connecting the pushing locations in both undistorted and distorted clips. TP: true pushing. FP: false pushing. TNP: true non-pushing. White circles: ground truth pushing. Red boxes: predicted pushing patches. Green boxes: predicted non-pushing patches.

A qualitative methodology was used in both evaluations; the methodology consisted of four steps: (1) we applied the framework to annotate distorted clips corresponding to MIMs in the RAFT-MIM${}^{Q_{12}}$-based test set (Figure 16); the bottom image is an example of an annotated distorted clip; (2) Unfortunately, we could not visualize the ground truth pushing on the distorted frames because the trajectory data were inaccurate. Therefore, we visualized ground truth pushing on the first frame of the corresponding undistorted clips to the distorted clips, Figure 16, top image. Then, we manually identified pushing behaviors on the distorted clips based on the corresponding annotated undistorted clips; This process is highlighted by arrows in Figure 16. (3) We manually calculated the number of true pushing, false pushing, true non-pushing, and false non-pushing. Note that the empty patches were discarded. Non-empty patches containing more than half of the pushing behaviors are labeled as pushing; otherwise, they are labeled as non-pushing. Half of the pushing behavior means that more than half of the visible pedestrian body contributes to pushing; (4) Finally, we measured the accuracy and F1 score metrics.

From Table 9, we can see that our framework with the false reduction algorithm can achieve an 86% accuracy and F1 score on the distorted videos. Moreover, the false reduction improves the performance by 2%.

## 6. Conclusions, Limitations, and Future Work

This paper proposed a hybrid deep learning and visualization framework for automatic pushing behavior detection at the patch level, particularly from top-view video recordings of crowded event entrances. The framework mainly relied on the power of EfficientNet-B0-based CNN, RAFT, and wheel visualization methods to overcome the high complexity of pushing behavior detection. RAFT and wheel visualization are combined to extract crowd motion information and generate MIM patches. After that, the combination of the EfficientNet-B0-based classifier and false reduction algorithm detects the pushing MIM patches and produces the pushing annotated video. In addition to the proposed framework, we introduced an efficient patch-based approach to increase the number of samples and alleviate the class imbalance issue in pushing datasets. The approach aims to improve the accuracy of the classifier and the proposed framework. Furthermore, we created new datasets using a real-world ground truth of pushing behavior videos and the proposed patch-based approach for evaluation. The experimental results show that: (1) the patch-based medium RAFT-MIM${}^{Q_{12}}$ dataset is the best compared to the other generated datasets for training the CNN-based classifiers; (2) Our classifier outperformed the baseline well-known CNN architectures in image classification as well as customized CNN architectures in the related works; (3) Compared to Farnebäck, RAFT improved the accuracy of the proposed classifier by 8%; (4) The proposed patch-based approach helped to enhance our classifier accuracy from 78% to 88%; (5) Overall, the proposed adapted EfficientNet-B0-based classifier obtained 88% accuracy on the patch-based medium RAFT-MIM${}^{Q_{12}}$ dataset; (6) The above results were based on undistorted videos, while the proposed framework obtained 86% accuracy on the distorted videos; (7) The developed false reduction algorithm improved the framework accuracy on distorted videos from 84% to 86%. The main reason behind decreasing the framework accuracy on distorted videos was training the classifier based on undistorted videos.

The main limitations of the proposed framework cannot be applied in real time. Additionally, it does not work well with recorded videos from a moving camera. Moreover, the framework was evaluated only on specific scenarios of crowded event entrances.

In future work, we plan to evaluate our framework in more scenarios of crowded event entrances. Additionally, we plan to optimize the proposed framework to allow real-time detection.

## Figures and Tables

**Table 1:** Characteristics of the selected experiments.

Experiment *	Width (m)	Pedestrians	Direction	Frames **
110	1.2	63	Left to right	1285
150	5.6	57	Left to right	1408
170	1.2	25	Left to right	552
270	3.4	67	Right to left	1430
280	3.4	67	Right to left	1640

* The same names as reported in [42]; ** The number of frames that contain pedestrians in the ROI.

**Table 2:** Number of labeled samples in training, validation, and test sets for each MIM-based dataset.

		Experiment												
Dataset		110		150		170		270		280		All		
		P	NP	P	NP	P	NP	P	NP	P	NP	P	NP	Total
	Training	66	16	76	14	28	5	61	29	86	11	317	75	392
RAFT-MIM${}^{Q_{12}}$	Validation	13	3	15	3	5	1	13	6	18	2	64	15	79
	Test	13	3	15	3	5	1	13	6	18	2	64	15	79
	Total	92	22	106	20	38	7	87	41	122	15	445	105	550
	Training	30	6	35	6	13	1	29	13	40	4	147	30	177
RAFT-MIM${}^{Q_{25}}$	Validation	6	2	7	1	3	1	6	2	8	1	30	7	37
	Test	6	2	7	1	3	1	6	2	8	1	30	7	37
	Total	42	10	49	8	19	3	41	17	56	6	207	44	251
Farnebäck-MIM${}^{Q_{12}}$	It has the same samples as the RAFT${}^{Q_{12}}$ sets while they are generated using Farnebäck.													
Farnebäck-MIM${}^{Q_{25}}$	It has the same samples as the RAFT${}^{Q_{25}}$ sets while they are generated using Farnebäck.													

P: pushing samples. NP: non-pushing samples. All: all experiments. 110, 150, 170, 270, and 280: names of the video experiments.

**Table 3:** Number of labeled MIM patches in training and validation sets for each patch-based MIM dataset.

		Experiment												
Dataset		110		150		170		270		280		All		
		P	NP	P	NP	P	NP	P	NP	P	NP	P	NP	Total
	Training	350	279	523	932	121	97	528	784	634	806	2156	2898	5054
Patch-based small RAFT-MIM${}^{Q_{12}}$	Validation	67	53	89	161	20	21	91	169	108	162	375	566	941
	Total	417	332	612	1093	141	118	619	953	742	968	2531	3464	5995
	Training	156	124	249	419	53	42	236	379	324	354	1018	1318	2336
Patch-based small RAFT-MIM${}^{Q_{25}}$	Validation	33	26	35	82	9	12	56	53	67	89	200	262	462
	Total	189	150	284	501	62	54	292	432	391	443	1218	1580	2798
	Training	237	131	298	354	95	38	540	439	698	326	1868	1288	3156
Patch-based medium RAFT-MIM${}^{Q_{12}}$	Validation	45	26	55	64	16	8	98	105	126	81	340	284	624
	Total	282	157	353	418	111	46	638	544	824	407	2208	1572	3780
	Training	107	58	142	151	42	14	242	219	338	146	871	585	1459
Patch-based medium RAFT-MIM${}^{Q_{25}}$	Validation	22	14	20	37	8	6	56	27	68	32	174	116	290
	Total	129	72	162	188	50	20	298	246	406	178	1045	704	1749

P: pushing samples. NP: non-pushing samples. All: all experiments. 110, 150, 170, 270, and 280: names of the video experiments.

**Table 4:** Number of labeled MIM patches in patch-based test sets.

	Experiment												
Test Set	110		150		170		270		280		All		
	P	NP	P	NP	P	NP	P	NP	P	NP	P	NP	Total
Patch-based small RAFT-MIM${}^{Q_{12}}$ test	40	28	47	99	9	13	59	112	61	108	216	360	576
Patch-based small RAFT-MIM${}^{Q_{25}}$ test	18	15	19	44	7	8	28	54	25	36	97	157	254
Patch-based medium RAFT-MIM${}^{Q_{12}}$ test	26	16	25	47	8	6	47	41	50	40	156	150	306
Patch-based medium RAFT-MIM${}^{Q_{25}}$ test	13	8	8	26	5	5	22	19	20	18	68	76	144

P: pushing samples. NP: non-pushing samples. All: all experiments. 110, 150, 170, 270, and 280: names of the video experiments.

**Table 5:** Comparison with well-known CNN-based classifiers on patch-based MIM datasets.

	Patch-Based MIM Dataset							
CNN-Based Classifier	Medium RAFT-MIM${}^{Q_{12}}$		Small RAFT-MIM${}^{Q_{12}}$		Medium RAFT-MIM${}^{Q_{25}}$		Small RAFT-MIM${}^{Q_{25}}$	
	Accuracy%	F1 Score%	Accuracy%	F1 Score%	Accuracy%	F1 Score%	Accuracy%	F1 Score%
MobileNet	87	87	79	78	85	85	77	74
EfficientNet-B0	**88**	**88**	**81**	**80**	**87**	**87**	**78**	**78**
InceptionV3	85	85	76	75	80	80	76	74
ResNet50	80	80	70	70	74	73	71	69

Bold: best results in each dataset. Gray highlight: Best results among all datasets.

**Table 6:** MIM -based classifier evaluation.

	Patch-Based Classifier		MIM-Based Classifier	
**CNN-Based Classifier**	**Accuracy%**	**F1 Score%**	**Accuracy%**	**F1 Score%**
MobileNet	87	87	71	69
EfficientNet-B0	88	88	78	78
InceptionV3	85	85	51	34
ResNet50	80	80	51	34

**Table 7:** Comparison between RAFT-based classifiers and Farnebäck-based classifiers.

	RAFT-Based Classifier		Farnebäck-Based Classifier	
**Classifier**	**Accuracy%**	**F1 Score%**	**Accuracy%**	**F1 Score%**
MobileNet	87	87	81	81
EfficientNet-B0	88	88	80	80
InceptionV3	85	85	79	79
ResNet50	80	80	74	73

**Table 8:** Comparisons to the customized CNN-based classifiers in the related works.

Classifier	Accuracy%	F1 Score%
EfficientNet-B0 (our classifier)	88	88
CNN-1 [25]	60	54
CNN-2 [35]	54	35

**Table 9:** The performance of the framework with and without false reduction on distorted videos.

Framework	Accuracy%	F1 Score%
Without false reduction	84	84
With false reduction	86	86

## Data Availability

All videos and trajectory data used in generating the datasets were obtained from the data archive hosted by the Forschungszentrum Jülich under CC Attribution 4.0 International license [42]. The undistorted videos, trained CNN-based classifiers, test sets, results, codes (framework; building, training and evaluating the classifiers) generated or used in this paper are publicly available at: https://github.com/PedestrianDynamics/DL4PuDe (accessed on 10 April 2022). The training and validation sets are available from the author upon request.

## References

[1] Adrian J., Boltes M., Sieben A., Seyfried A. (2020). Influence of Corridor Width and Motivation on Pedestrians in Front of Bottlenecks. Traffic and Granular Flow 2019.

[2] Adrian J., Seyfried A., Sieben A. (2020). Crowds in front of bottlenecks at entrances from the perspective of physics and social psychology. J. R. Soc. Interface.

[3] Lügering H., Üsten E., Sieben A. (2022). Pushing and Non-Pushing Forward Motion in Crowds: A Systematic Psychological Method for Rating Individual Behavior in Pedestrian Dynamics.

[4] Haghani M., Sarvi M., Shahhoseini Z. (2019). When ‘push’does not come to ‘shove’: Revisiting ‘faster is slower’in collective egress of human crowds. Transp. Res. Part A Policy Pract..

[5] Sieben A., Schumann J., Seyfried A. (2017). Collective phenomena in crowds—Where pedestrian dynamics need social psychology. PLoS ONE.

[6] Adrian J., Boltes M., Holl S., Sieben A., Seyfried A. (2018). Crowding and queuing in entrance scenarios: Influence of corridor width in front of bottlenecks. arXiv.

[7] Boltes M., Seyfried A., Steffen B., Schadschneider A. (2010). Automatic extraction of pedestrian trajectories from video recordings. Pedestrian and Evacuation Dynamics 2008.

[8] Nayak R., Pati U.C., Das S.K. (2021). A comprehensive review on deep learning-based methods for video anomaly detection. Image Vis. Comput..

[9] Roshtkhari M.J., Levine M.D. (2013). An on-line, real-time learning method for detecting anomalies in videos using spatio-temporal compositions. Comput. Vis. Image Underst..

[10] Singh G., Khosla A., Kapoor R. (2019). Crowd escape event detection via pooling features of optical flow for intelligent video surveillance systems. Int. J. Image Graph. Signal Process..

[11] George M., Bijitha C., Jose B.R. Crowd panic detection using autoencoder with non-uniform feature extraction. Proceedings of the 8th International Symposium on Embedded Computing and System Design (ISED).

[12] Santos G.L., Endo P.T., Monteiro K.H.D.C., Rocha E.D.S., Silva I., Lynn T. (2019). Accelerometer-based human fall detection using convolutional neural networks. Sensors.

[13] Mehmood A. (2021). LightAnomalyNet: A Lightweight Framework for Efficient Abnormal Behavior Detection. Sensors.

[14] Zhang X., Zhang Q., Hu S., Guo C., Yu H. (2018). Energy level-based abnormal crowd behavior detection. Sensors.

[15] Kooij J.F., Liem M.C., Krijnders J.D., Andringa T.C., Gavrila D.M. (2016). Multi-modal human aggression detection. Comput. Vis. Image Underst..

[16] Gan H., Xu C., Hou W., Guo J., Liu K., Xue Y. (2022). Spatiotemporal graph convolutional network for automated detection and analysis of social behaviours among pre-weaning piglets. Biosyst. Eng..

[17] Gan H., Ou M., Huang E., Xu C., Li S., Li J., Liu K., Xue Y. (2021). Automated detection and analysis of social behaviors among preweaning piglets using key point-based spatial and temporal features. Comput. Electron. Agric..

[18] Vu T.H., Boonaert J., Ambellouis S., Taleb-Ahmed A. (2021). Multi-Channel Generative Framework and Supervised Learning for Anomaly Detection in Surveillance Videos. Sensors.

[19] Yamashita R., Nishio M., Do R.K.G., Togashi K. (2018). Convolutional neural networks: An overview and application in radiology. Insights Imaging.

[20] Li L., Zhang S., Wang B. (2021). Apple leaf disease identification with a small and imbalanced dataset based on lightweight convolutional networks. Sensors.

[21] Wang P., Fan E., Wang P. (2021). Comparative analysis of image classification algorithms based on traditional machine learning and deep learning. Pattern Recognit. Lett..

[22] Duman E., Erdem O.A. (2019). Anomaly detection in videos using optical flow and convolutional autoencoder. IEEE Access.

[23] Farnebäck G. Two-frame motion estimation based on polynomial expansion. Proceedings of the 13th Scandinavian Conference on Image Analysis.

[24] Ilyas Z., Aziz Z., Qasim T., Bhatti N., Hayat M.F. (2021). A hybrid deep network based approach for crowd anomaly detection. Multimed. Tools Appl..

[25] Direkoglu C. (2020). Abnormal crowd behavior detection using motion information images and convolutional neural networks. IEEE Access.

[26] Almazroey A.A., Jarraya S.K. Abnormal Events and Behavior Detection in Crowd Scenes Based on Deep Learning and Neighborhood Component Analysis Feature Selection. Proceedings of the International Conference on Artificial Intelligence and Computer Vision (AICV2020).

[27] Teed Z., Deng J. Raft: Recurrent all-pairs field transforms for optical flow. Proceedings of the European Conference on Computer Vision.

[28] Tom Runia D.F. Optical Flow Visualization. https://github.com/tomrunia/OpticalFlow_Visualization.

[29] Baker S., Scharstein D., Lewis J., Roth S., Black M.J., Szeliski R. (2011). A database and evaluation methodology for optical flow. Int. J. Comput. Vis..

[30] Tan M., Le Q. Efficientnet: Rethinking model scaling for convolutional neural networks. Proceedings of the International Conference on Machine Learning.

[31] Deng J., Dong W., Socher R., Li L.J., Li K., Fei-Fei L. Imagenet: A large-scale hierarchical image database. Proceedings of the 2009 IEEE Conference on Computer Vision and Pattern Recognition.

[32] Coşar S., Donatiello G., Bogorny V., Garate C., Alvares L.O., Brémond F. (2016). Toward abnormal trajectory and event detection in video surveillance. IEEE Trans. Circuits Syst. Video Technol..

[33] Jiang J., Wang X., Gao M., Pan J., Zhao C., Wang J. (2022). Abnormal behavior detection using streak flow acceleration. Appl. Intell..

[34] Xu M., Yu X., Chen D., Wu C., Jiang Y. (2019). An efficient anomaly detection system for crowded scenes using variational autoencoders. Appl. Sci..

[35] Tay N.C., Connie T., Ong T.S., Goh K.O.M., Teh P.S. (2019). A robust abnormal behavior detection method using convolutional neural network. Computational Science and Technology.

[36] Sabokrou M., Fayyaz M., Fathy M., Moayed Z., Klette R. (2018). Deep-anomaly: Fully convolutional neural network for fast anomaly detection in crowded scenes. Comput. Vis. Image Underst..

[37] Smeureanu S., Ionescu R.T., Popescu M., Alexe B. Deep appearance features for abnormal behavior detection in video. Proceedings of the International Conference on Image Analysis and Processing.

[38] Khan S.S., Madden M.G. (2014). One-class classification: Taxonomy of study and review of techniques. Knowl. Eng. Rev..

[39] Hu Y. (2020). Design and implementation of abnormal behavior detection based on deep intelligent analysis algorithms in massive video surveillance. J. Grid Comput..

[40] Zhou S., Shen W., Zeng D., Fang M., Wei Y., Zhang Z. (2016). Spatial–temporal convolutional neural networks for anomaly detection and localization in crowded scenes. Signal Process. Image Commun..

[41] Zhang C., Xu Y., Xu Z., Huang J., Lu J. (2022). Hybrid handcrafted and learned feature framework for human action recognition. Appl. Intell..

[42] Adrian J., Seyfried A., Sieben A. Crowds in Front of Bottlenecks from the Perspective of Physics and Social Psychology. http://ped.fz-juelich.de/da/2018crowdqueue.

[43] Hollows G., James N. (2016). Understanding Focal Length and Field of View. Retrieved Oct..

[44] Srivastava N., Hinton G., Krizhevsky A., Sutskever I., Salakhutdinov R. (2014). Dropout: A simple way to prevent neural networks from overfitting. J. Mach. Learn. Res..

[45] Genc B., Tunc H. (2019). Optimal training and test sets design for machine learning. Turk. J. Electr. Eng. Comput. Sci..

[46] Ismael S.A.A., Mohammed A., Hefny H. (2020). An enhanced deep learning approach for brain cancer MRI images classification using residual networks. Artif. Intell. Med..

[47] Howard A.G., Zhu M., Chen B., Kalenichenko D., Wang W., Weyand T., Andreetto M., Adam H. (2017). Mobilenets: Efficient convolutional neural networks for mobile vision applications. arXiv.

[48] Szegedy C., Vanhoucke V., Ioffe S., Shlens J., Wojna Z. Rethinking the inception architecture for computer vision. Proceedings of the IEEE Conference on Computer Vision and Pattern Recognition.

[49] He K., Zhang X., Ren S., Sun J. Deep residual learning for image recognition. Proceedings of the IEEE Conference on Computer Vision and Pattern Recognition.

[50] Van der Jeught S., Buytaert J.A., Dirckx J.J. (2012). Real-time geometric lens distortion correction using a graphics processing unit. Opt. Eng..

[51] Stankiewicz O., Lafruit G., Domański M. (2018). Multiview video: Acquisition, processing, compression, and virtual view rendering. Academic Press Library in Signal Processing.

[52] Vieira L.H., Pagnoca E.A., Milioni F., Barbieri R.A., Menezes R.P., Alvarez L., Déniz L.G., Santana-Cedrés D., Santiago P.R. (2017). Tracking futsal players with a wide-angle lens camera: Accuracy analysis of the radial distortion correction based on an improved Hough transform algorithm. Comput. Methods Biomech. Biomed. Eng. Imaging Vis..

